# Elasticity and glocality: initiation of embryonic inversion in *Volvox*

**DOI:** 10.1098/rsif.2015.0671

**Published:** 2015-11-06

**Authors:** Pierre A. Haas, Raymond E. Goldstein

**Affiliations:** Department of Applied Mathematics and Theoretical Physics, Centre for Mathematical Sciences, University of Cambridge, Wilberforce Road, Cambridge CB3 0WA, UK

**Keywords:** cell sheet folding, embryonic inversion, *Volvox*

## Abstract

Elastic objects across a wide range of scales deform under local changes of their intrinsic properties, yet the shapes are *glocal*, set by a complicated balance between local properties and global geometric constraints. Here, we explore this interplay during the inversion process of the green alga *Volvox*, whose embryos must turn themselves inside out to complete their development. This process has recently been shown to be well described by the deformations of an elastic shell under local variations of its intrinsic curvatures and stretches, although the detailed mechanics of the process have remained unclear. Through a combination of asymptotic analysis and numerical studies of the bifurcation behaviour, we illustrate how appropriate local deformations can overcome global constraints to initiate inversion.

## 1. Introduction

The shape of many a deformable object arises through the competition of multiple constraints on the object: this competition may be between different global constraints, such as in Helfrich's analysis [1] of the shape of a red blood cell (where intrinsic curvature effects coexist with constrained membrane area and enclosed volume). It may also be the competition between local and global constraints. Such deformations, which we shall term *glocal*, arise for example in origami patterns [2] (where local folds must be compatible with the global geometry). They are of considerable interest in the design of programmable materials [3] at macro- and microscales, where one asks: can a sequence of local deformations overcome global constraints and direct the global deformations of an object?

**Figure 1.**
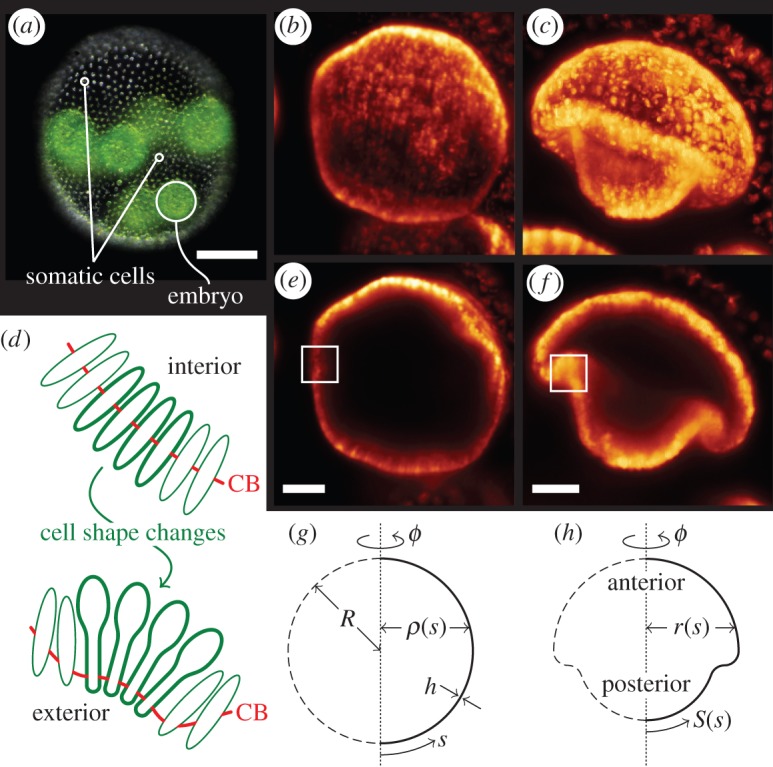
*Volvox* invagination and elastic model. (*a*) Adult *Volvox*, with somatic cells and one embryo labelled. (*b*) *Volvox* embryo at the start of inversion. (*c*) Mushroom-shaped invaginated *Volvox* embryo. (*d*) Cell shape changes to wedge shapes and motion of cytoplasmic bridges (CB) bend the cell sheet. Red line indicates position of cytoplasmic bridges. (*e*,*f*) Cross sections of the stages shown in panels (*b*,*c*). Cell shape changes as in (*d*) occur in the marked regions. (*g*) Geometry of undeformed spherical shell of radius *R* and thickness *h*. (*h*) Geometry of the deformed shell. Scale bars: (*a*) 50 µm, (*e*,*f*) 20 µm. False colour images obtained from light-sheet microscopy provided by Stephanie Höhn and Aurelia R. Honerkamp-Smith.

This is a problem that, at the close of their development, the embryos of the green alga *Volvox* [4] are faced with in the ponds of this world. *Volvox* (figure 1*a*) is a multicellular green alga belonging to a lineage (the Volvocales) that has been recognized since the time of Weismann [5] as a model organism for the evolution of multicellularity, and which more recently has emerged as the same for biological fluid dynamics [6]. The Volvocales span from unicellular *Chlamydomonas*, through organisms such as *Gonium*, consisting of 8 or 16 *Chlamydomonas*-like cells in a quasi-planar arrangement, to spheroidal species (*Pandorina* and *Pleodorina*) with scores or hundreds of cells at the surface of a transparent extracellular matrix (ECM). The largest members of the Volvocales are the species of *Volvox*, which display germ–soma differentiation, having sterile somatic cells at the surface of the ECM and a small number of germ cells in the interior which develop to become the daughter colonies.

Following a period of substantial growth, the germ cells of *Volvox* undergo repeated rounds of cell division, at the end of which each embryo (figure 1*b,e*) consists of a few thousand cells arrayed to form a thin spherical sheet [4]. These cells are connected to each other by the remnants of incomplete cell division, thin membrane tubes called *cytoplasmic bridges* [7,8]. The ends of the cells whence emanate the flagella, however, point into the sphere at this stage, and so the ability to swim is only acquired once the alga turns itself inside out through an opening at the top of the cell sheet, called the *phialopore* [9–11].

Of particular interest in the present context is the crucial first step of this process, the formation of a circular invagination in so-called type B inversion (figure 1*c*,*f*) followed by the engulfing of the posterior by the anterior hemisphere [11,12]. (This scenario is distinct from ‘type A’ inversion in which the initial steps involve four lips which peel back from a cross-shaped phialopore [11].) The invaginations of cell sheets found in type B inversion are very generic deformations during morphogenetic events such as gastrulation and neurulation [13–16], but, in animal model organisms, they often arise from an intricate interplay of cell division, intercalation, migration and cell shape changes. For this reason, descriptions thereof have ofttimes invoked cell-based models, as pioneered by Odell *et al.* [17], but simpler models of simpler morphogenetic processes are required to elucidate the underlying mechanics of these problems [18]. Inversion in *Volvox* is, however, driven by active cell shape changes alone: inversion starts when cells close to the equator of the shell elongate and become wedge-shaped [12]. Simultaneously, the cytoplasmic bridges migrate to the wedge ends of the cells, thus splaying the cells locally and causing the cell sheet to bend [12] (figure 1*d*). Additional cell shape changes have been implicated in the relative contraction of one hemisphere with respect to the other in order to facilitate invagination [19]. Examination of thin sections suggests that, as invagination progresses, more cells in the posterior hemisphere change their shape, but the cells in the bend region are less markedly wedge-shaped after invagination [12]. As inversion progresses further, the bend region continues to expand, allowing the posterior hemisphere to invert fully, eventually expanding into the anterior hemisphere, too.

At a more physical level, it has been shown recently that the inversion process is simple enough to be amenable to a mathematical description [19]: the deformations of the alga are well reproduced by a simple elastic model in which the cell shape changes and motion of cytoplasmic bridges impart local variations of intrinsic curvature and stretches to an elastic shell [19]. This work raised, however, a host of more mechanical questions: how do intrinsic stretches and curvatures conspire with the global geometry of the shell? What mechanical regimes arise in this elastogeometric coven? What are the simple geometric balances that underlie these regimes and how do they relate to the biological problem? These questions we address in the present work. Using the elastic model introduced earlier [19], an asymptotic analysis at small deformations clarifies the geometric distinction between deformations resulting from intrinsic bending and intrinsic stretching, respectively. An in-depth study, both analytical and numerical, of the bifurcation behaviour over a broader parameter range than considered earlier [19] illustrates how a sequence of local deformations can achieve invagination, and how contraction complements bending in this picture.

## 2. Elastic model

Following Höhn *et al.* [19], we inscribe *Volvox* inversion into the very general framework of the axisymmetric deformations of a thin elastic spherical shell of radius *R* and thickness 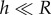 under variations of its intrinsic curvature and stretches. The undeformed, spherical, configuration of the shell is characterized by arclength *s* and the distance of the shell from its axis of revolution, *ρ*(*s*) (figure 1*g*). To these correspond arclength *S*(*s*) and distance from the axis of revolution *r*(*s*) in the deformed configuration (figure 1*h*). The undeformed and deformed configurations are related by the meridional and circumferential stretches,2.1
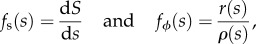
(These definitions do not require that the undeformed configuration be spherical, and apply for the deformations of any axisymmetric object.) These define the strains2.2
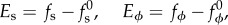
and curvature strains2.3
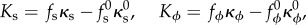
where *κ*_s_ and *κ*_*ϕ*_ denote the meridional and circumferential curvatures of the deformed shell. The intrinsic stretches and curvatures introduced by 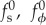 and 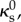 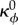 extend Helfrich's work on membranes [1]. The deformed configuration of the shell minimizes an energy of the Hookean form [20–22]2.4
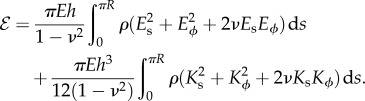
with material parameters the elastic modulus *E* and Poisson's ratio *ν*. In computations, we take *h*/*R* = 0.15 and *ν* = 1/2 appropriate for *Volvox* inversion [19]. Because the elastic modulus appears as a common prefactor to both contributions to the energy in (2.4), the equilibrium shapes found by minimizing 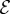 are independent of *E*. This reflects the fact that *E* only enters into the magnitude of the stresses within the shell.

In general, deformations of the shell arise from a complex interplay of intrinsic stretches and curvatures, and the global geometry of the shell. To clarify these, we begin by considering two simple kinds of deformations, in which the competition is between two effects only. How these effects conspire in general we shall explore in the main body of the paper.

### 2.1. Simple deformations: growing/shrinking and bending

**Figure 2.**
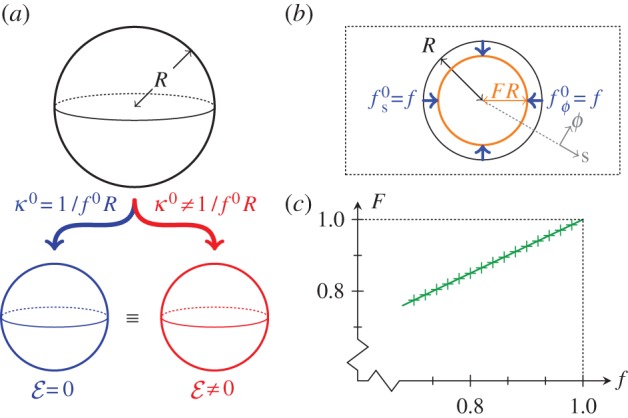
Simple intrinsic deformations. (*a*) A sphere can be shrunk to smaller spheres of equal radii by both compatible and incompatible intrinsic deformations. (*b*) Contraction of a circular region of radius *R* in a plane elastic sheet by a factor *f*. The boundary of this region is contracted to *s* = *FR*. (*c*) Numerical result for *F* (+) agrees with analytical calculation (2.9) (solid line).

The simplest intrinsic deformation is one of uniform stretching or contraction, which does not affect the global, spherical geometry of the shell. This corresponds to 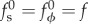 and 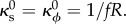 With these intrinsic stretches and curvatures, the original sphere deforms to a sphere of radius 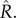 Then, 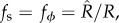 and so 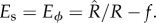 However, 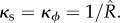 Thus 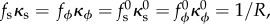 and hence 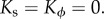 The energy density is therefore proportional to 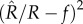 and is minimized for 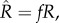 at which point 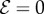 (figure 2*a*). (Indeed, uniform contraction is a homothetic transformation: the angles between material points are unchanged, and so there is no bending involved. In other words, the shell is blind to its intrinsic curvature on this spherical solution branch.)

The intrinsic stretches and curvatures need not be compatible in this way, however: suppose that 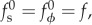 but 
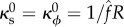
 with 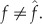 The energy still has spherical minima of radius 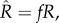 but now with 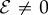 (figure 2*a*). This illustrates that, conversely, even if the equilibrium shape is spherical, the intrinsic curvatures and stretches cannot straightforwardly be inferred from the resulting shape.

### 2.2. Simple deformations: shrinking and geometry

To illustrate how the global geometry affects these deformations, we consider contraction of a plane elastic sheet, with 
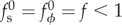
 for *s* < *R* (figure 2*b*). This is a version of the classical Lamé problem [23]: there is no bending of the sheet involved, and, upon non-dimensionalizing lengths with *R*, the sheet minimizes2.5
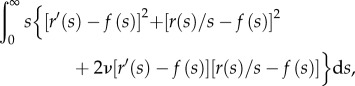
where2.6
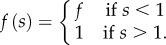
The resulting Euler–Lagrange equation is2.7
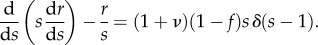
This is a homogeneous equation, and the solution satisfying the geometric conditions *r*(0) = 0 and *r*(*s*)∼*s* as 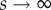 as well as continuity of *r* at *s* = 1 is2.8
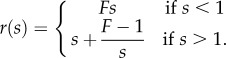
The constant *F* = *r*(1) is determined by the jump condition at *s* = 1, or, physically, by requiring the stress to be continuous across *s* = 1. This finally yields2.9
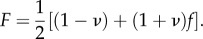
This simplified problem serves as a test case for numerical solution of the more general Euler–Lagrange equations associated with (2.4). These boundary-value problems can be solved numerically with the solver bvp4c of Matlab^®^ (The MathWorks, Inc.); our numerical set-up of the governing equations otherwise mimics that of Knoche & Kierfeld [22]. In this particular example, the linear relationship in (2.9) is indeed confirmed numerically (figure 2*c*). Note that, away from *s* = 1, the governing equation (2.7) is independent of the forcing applied; the solution is determined by geometric boundary conditions.

## 3. Results

The most drastic cell shape changes at the start of inversion occur when cells in a narrow region close to the equator become wedge-shaped (figure 1*d*). These are accompanied by motion of the cytoplasmic bridges to the thin tips of the cells to splay the cell sheet and drive its inward bending. For this reason, Höhn *et al.* [19] started by considering a piecewise constant functional form for the intrinsic curvature in which this curvature took negative values in a narrow region close to the equator. It was found, however, that with this ingredient alone the energy minimizers could not reproduce the mushroom shapes adopted by the embryos in the early of stages of inversion (figure 1*c*,*f*), producing instead a shape cinched in at those points—the so-called purse-string effect. However, analysis of thin sections had previously revealed that the cells in the posterior hemisphere become thinner at the start of inversion [12]. When the resulting contraction of the posterior hemisphere was incorporated into the model, it could indeed reproduce, quantitatively, the shapes of invaginating *Volvox* embryos.

Höhn *et al.* have thus identified two different types of active deformations that contribute to the shapes of inverting *Volvox* at the invagination stage: first, a localized region of active inward bending (corresponding to negative intrinsic curvature), and second, relative contraction of one hemisphere with respect to the other. We shall focus on these two types of deformation in what follows and clarify the ensuing elastic and geometrical balances.

### 3.1. Asymptotic analysis

**Figure 3.**
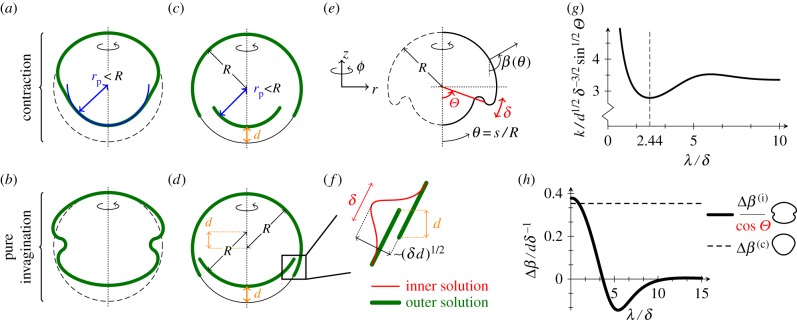
Asymptotic analysis of invagination and contraction. (*a*) Numerical shape resulting from contracting the posterior to a radius *r*_p_ < *R.* (*b*) Numerical ‘hourglass’ shape resulting from pure invagination. (*c*) Geometry of contraction with posterior radius *r*_p_ < *R*, resulting in upward motion of the posterior by a distance *d*. (*d*) Geometry of pure invagination solution. (*e*) Asymptotic geometry: in the limit 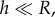 deformations are localized to an asymptotic inner layer of width *δ* about *θ* = *Θ*, where *θ* = *s*/*R* is the angle that the undeformed normal makes with the vertical. In the deformed configuration, this angle has changed to *β*(*θ*). (*f*) Asymptotic invagination: upward motion of the posterior by a distance *d* requires inward deformations scaling as (*δd*)^1/2^ in the inner layer of width *δ*. (*g*) Relation between preferred curvature *k* and width of invagination *λ* for a given amount of upward posterior motion *d*, from asymptotic calculations. (*h*) Inward rotation Δ*β* of the midpoint of the invagination with, and without contraction, from asymptotic calculations.

The (initially) narrow region of cell shape changes invites an asymptotic analysis. We therefore start by seeking equilibrium configurations in the limit of a thin shell, 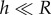. In this limit, the shapes (figure 3*a*,*b*) corresponding to contraction or (pure) invagination (by which we mean, here, deformations driven by a region of high intrinsic curvature only) result from the matching of spherical shells of different radii or disparate relative positions (figure 3*c*,*d*). Deviations from these outer solutions are localized to an asymptotic inner layer of non-dimensional width *δ* about *θ* = *Θ*, where *θ* = *s*/*R* is the angle that the normal to the undeformed shell makes with the vertical, i.e. the azimuthal angle of the undeformed shell, measured from the posterior pole (figure 3*e*). Here, we consider an incipient deformation where the normal angle *β*(*θ*) to the deformed shell deviates but slightly from its value in the spherical configuration, viz. 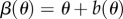, with 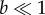.

#### 3.1.1. Geometric considerations

We begin by clarifying the geometric distinction between contraction and invagination. The radial and vertical displacements obey3.1*a*
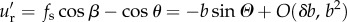
and3.1*b*
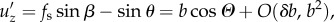
where dashes denote differentiation with respect to *θ*, and where we have assumed the scaling 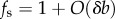 which follows from the detailed asymptotic solution. Let *d* denote the (non-dimensional) distance by which the posterior moves up. Matching to the outer solutions requires the net displacements *U*_r_ and *U_z_*, obtained by integrating (3.1) across the inner layer, to obey3.2*a*
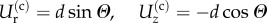
and3.2*b*
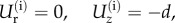
where the superscripts (c) and (i) refer, respectively, to the solutions corresponding to contraction and (pure) invagination. In the case of contraction, (3.1) and (3.2*a*) give the scaling *b*^(c)^ ∼ *d*/*δ*. If there is no contraction, however, (3.1) and (3.2*b*) imply that the leading-order solution does not yield any upward motion of the posterior, which is associated with a higher-order solution only. This suggests that the appropriate scaling is 
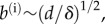
 which we shall verify presently.

Our assumption 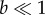 thus translates to 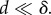 Hence, in the invagination case, upward motion of the posterior requires comparatively large inward displacements of order 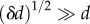 (figure 3*f*). This asymptotic difference of the deformations corresponding to contraction and invagination arises purely from geometric effects; it is the origin of the ‘purse-string’ shapes found by Höhn *et al.* [19] in the absence of contraction.

#### 3.1.2. Elastogeometric considerations

The detailed asymptotic solution for pure invagination is somewhat involved. We refer to appendix B for the details of the calculation, and, here, summarize the results, with distances non-dimensionalized with *R*.

The pure invagination configuration is forced by intrinsic curvature that differs from the curvature of the undeformed sphere in a region of width *λ* about *θ* = *Θ*, where 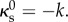 Scaling reveals that 
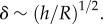
 Writing 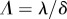 and 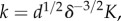 a long calculation leads to3.3
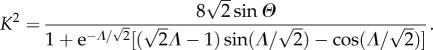


This function exhibits a global minimum at 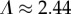 (figure 3*g*). This is a first indication that narrow invaginations are more efficient than those resulting from wider regions of high intrinsic curvature, a statement that we shall make more precise later.

Symmetry implies that there is no inward rotation of the midpoint of the invagination at this order. Rather, inward folding is a second-order effect, and the second-order problem implies that the rotation of the midpoint of the invagination is3.4
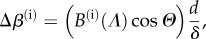
where 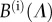 is determined by the detailed solution of said second-order problem. The geometric factor in (3.4) is, however, the main point: this factor resulting from the global geometry of the shell hampers the inward rotation of the midpoint of the invagination. (This is as expected: by symmetry, invagination at the equator, where cos *Θ* = 0, yields no rotation.)

An analogous, though considerably more straightforward calculation, can be carried out for contraction: non-dimensionally, upward posterior motion by *d* requires 
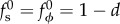
 for *θ* < *Θ*, and leads to3.5
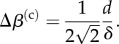


At this order, the above solutions for pure invagination and contraction can be superposed. For contraction, there is thus no geometric obstacle to inward folding (figure 3*h*). Hence, contraction is not only a means of creating the disparity in the radii of the anterior and posterior hemispheres required to fit the partly inverted latter into the former, but also drives the inward folding of the invagination, by breaking its symmetry. In *Volvox* inversion, this symmetry breaking is at the origin of the formation of the second passive bend region highlighted by Höhn *et al.* [19] to stress the non-local character of these deformations.

### 3.2. Bifurcation behaviour

**Figure 4.**
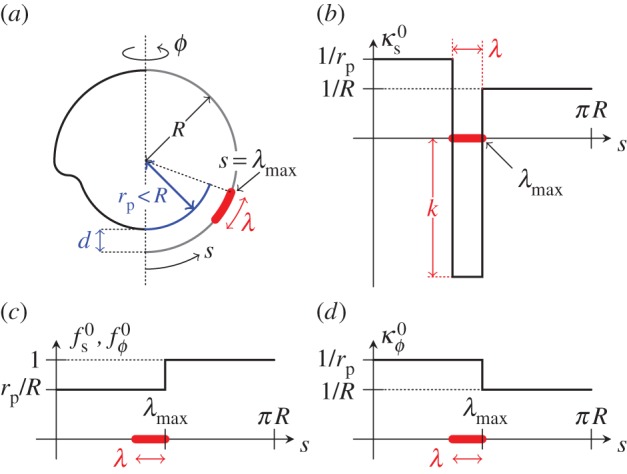
Set-up for numerical calculations, following Höhn *et al*. [19]. (*a*) Geometrical set-up: the intrinsic curvature 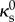 of a spherical shell of undeformed radius *R* differs from the undeformed curvature in the arclength range *λ*_max_ > *s* > *λ*_max_ − *λ*, where *s* is arclength. Posterior contraction is taken into account by a reduced posterior radius *r*_p_ < *R*. These intrinsic curvature and contraction result in deformations that move up the posterior pole by a distance *d*. (*b*) Corresponding functional form of 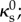 in the bend region, 
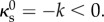
 (*c*) Form of the intrinsic stretches 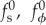 for posterior contraction. (*d*) Functional form of 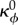 for posterior contraction.

The asymptotic analysis has shown that the coupling of elasticity and geometry constrains small invagination-like deformations both locally and globally, but that contraction can help overcome these global constraints. These ideas carry over to larger deformations of the shell, which must however be studied numerically. For this purpose, we extend the set-up of Höhn *et al*. [19], motivated by direct observation of thin sections of fixed embryos: the intrinsic curvature 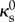 differs from that of undeformed sphere in the range *λ*_max_ > *s* > *λ*_max_ − *λ* of arclength along the shell (figure 4*a*). In this region of length *λ*, 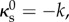 where *k* > 0 (figure 4*b*). This imposed intrinsic curvature results in upward motion of the posterior pole by a distance *d*.

**Figure 5.**
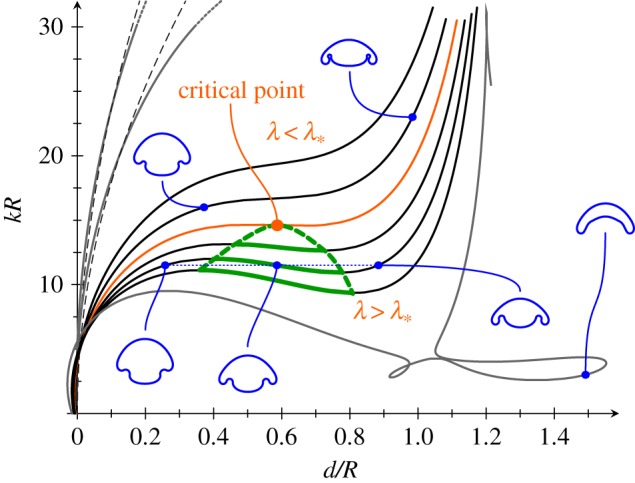
Bifurcation behaviour of invagination solutions. Solution space for *λ*_max_ = 1.1*R* and *r*_p_ = *R*: each line shows the relation between *k* and *d* at some constant *λ*. A critical branch (at *λ* = λ_*_) separates different types of branches. Branches with *λ* > λ_*_ feature two extrema; the resulting spinodal curve (thick dashed line) defines a critical point. Insets illustrate representative solution shapes. For branches with 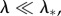 the asymptotic prediction 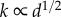 (thin dashed line) is recovered. See text for further explanation.

Our first observation is that, at fixed *λ*_max_, more than one solution may arise for the same input parameters (*k*, *λ*). Further understanding is gained by considering, at fixed *λ*_max_ and for different values of *λ*, the relation between *k* and *d*. The typical behaviour of these branches is plotted in figure 5. (The shapes eventually self-intersect; accordingly, these branches end, but we expect them to be joined up smoothly to configurations with opposite sides of the shell in contact. The study of such contact configurations typically requires some simplifying assumptions to be made [22], but we do not pursue this further, here.)

At the distinguished value *λ* = λ_*_, a critical branch arises (figure 5). It separates two types of branches: first, those with *λ* < λ_*_, on which *k* varies mononotonically with *d*, and second, those with *λ* > λ_*_, where the relation between *d* and *k* is more complicated. At large values of *λ*, these branches may have a rather involved topology involving loops. At values of *λ* just above λ_*_, however, there is a range of values of *k* for which there exist three configurations (figure 5). We note that the two outer configurations have 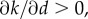 whereas the middle one has 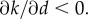 The latter behaviour prefigures instability, which we shall discuss in more detail below. There are thus two points on these branches where *k*, viewed locally as a function of *d*, reaches an extremum. The curve joining up these extrema for different values of *λ* we shall, borrowing thermodynamic nomenclature [24], term the ‘spinodal curve’. This curve, in turn, has a maximum at a point on the critical branch, which we shall call the ‘critical point’ and which is characterized by λ_*_ and the critical curvature, k_*_.

To make contact with the asymptotic calculations, we note that, for branches with 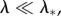 the proportionality 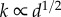 (at constant *λ*) is indeed recovered. The asymptotic result, formally valid in the range 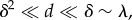 does not however capture the bifurcation behaviour.

#### 3.2.1. Effective energy

It is natural to ask whether this bifurcation can be understood in terms of an effective energy of some kind, balancing different elastogeometric effects. Here, we discuss the physical origin of the effects contributing to this effective energy. The details of the geometric approximations we leave to appendix C.

**Figure 6.**
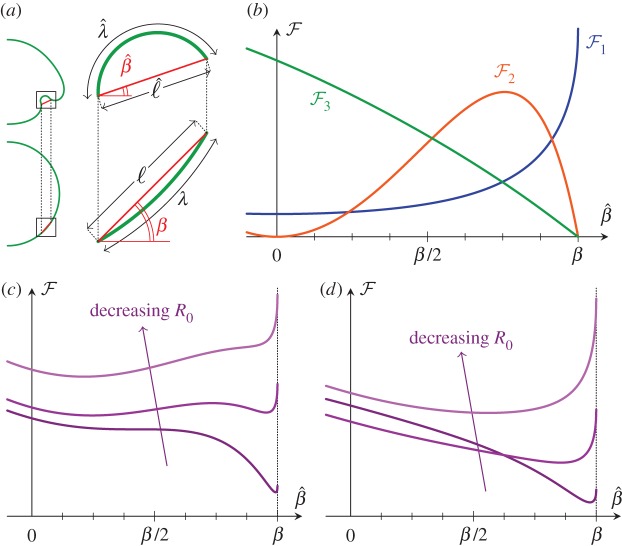
Effective energy underlying bifurcation behaviour. (*a*) Geometry of deformation and rotation of active bend region. (*b*) Functional forms of the contributions 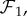 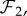 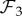 to the effective energy. (*c*) Effective energy landscape: two minima exist in an intermediate range of *R*_0_ at large enough *ℓ*. (*d*) The intermediate regime disappears if *ℓ* is small enough.

In the undeformed configuration and in a plane containing the axis of revolution of the shell, the active bend region is a circular arc of length *λ* and radius *R*, intercepting a chord of length 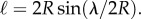 This chord makes an angle *β* with the radial outward direction (figure 6*a*). In the deformed configuration, this region has deformed to an arc of length 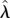 and radius 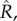 intercepting a chord of length 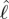 that makes an angle 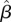 with the radial outward direction.

Stretching is energetically more costly than bending; at leading order, the meridional and circumferential strains must therefore vanish, thus3.6
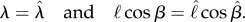
This allows the deformations of the shell to be described in terms of a single parameter.

Three effects contribute to the effective energy 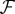: (i) the curvature 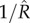 of the bend region, which may differ from its preferred curvature 1/*R*_0_, (ii) the additional hoop strain generated by the inward folding of the bend region and (iii) the formation of a second passive bend region owing to the inward folding of the first bend region. The functional forms of these contributions are derived in appendix C and shown in figure 6*b*.

Let 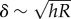 denote the bending lengthscale of the shell. In the limit 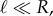 the scalings of the three terms contributing to the energy are found to be3.7
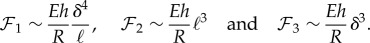


Accordingly, all three terms contribute to the energy when 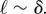 In this regime, but a single minimum exists in the energy landscape at large or small values of the intrinsic curvature, whereas two minima exist for intermediate values (figure 6*c*). If 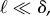 the second term is negligible, and the intermediate regime ceases to exist (figure 6*d*), as expected from the functional form of this term (figure 6*b*).

Conversely, this shows that this behaviour cannot be reproduced if any one of these three contributions is omitted from the analysis. If 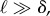 the third term is negligible, and the second minimum is predicted to exist even at low values of the intrinsic curvature. This disagrees with the behaviour found numerically, and so the approximations break down in this limit, as expected given the more exotic topologies that arise at large *λ* in figure 5.

The bifurcation behaviour can also be rationalized in terms of the (asymptotic) existence of geometrically ‘preferred’ modes that solve the equations expressing the leading asymptotic balance at large rotations in the absence of forcing (through intrinsic curvature or otherwise). We discuss this point further in appendix B.

#### 3.2.2. Stability statements

The stability of the configurations in figure 5 can be assessed by means of general results of bifurcation theory [25], used recently to discuss the stability of the buckled equilibrium shapes of a pressurized elastic spherical shell [22,26]. If we let 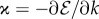 denote the conjugate variable to *k*, the key result of Maddocks [25] is that stability, at fixed *λ*, of extremizers of the energy 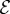 can be assessed from the folds in the 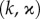 bifurcation diagram. In particular, stability can only change at folds in the bifurcation diagram. Expanding the bending part of the energy functional (2.4) for 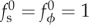 and 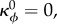 we find3.8
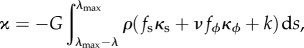
with 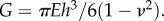 (The last term in the integrand is independent of the solution, and may therefore be ignored in what follows.)

**Figure 7.**
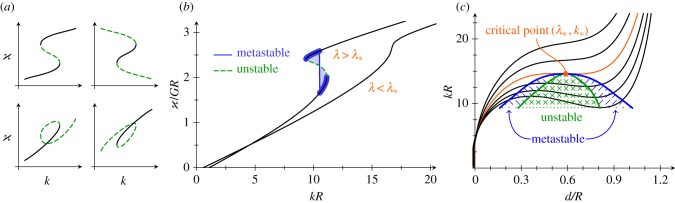
Stability of invagination solutions. (*a*) Possible topologies of a double fold in the distinguished 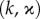 bifurcation diagram. Dashed branches are those that the results of Maddocks [25] imply to be unstable. (*b*) For *λ* > λ_*_, S-shaped folds arise in the 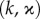 diagram. From general theory [25], the middle part of the branch is unstable, while the outer parts are stable. An additional region of metastability is identified by the Maxwell construction. (*c*) Resulting picture: a region of unstable and metastable solutions expands underneath the critical point.

The two folds that arise in the (*k*,*d*) diagram for *λ* > λ_*_ (figure 5) are compatible *a priori* with four fold topologies in the 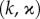 diagram (figure 7*a*). However, because a single solution exists for small *k* (at fixed *λ*), the lowest branch must be stable. Further, because the branches do not self-intersect in the (*k*, *d*) diagram, they cannot self-intersect in the 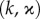 diagram either. The results of Maddocks [25] imply that only the first topology in figure 7*a* is compatible with this, and so the fold is S-shaped and traversed upwards in the 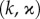 diagram. (Numerically, one confirms that the branches are indeed S-shaped.) It follows in particular that the middle branch, with 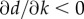 is unstable, and the right branch is stable (for it is the sole unstable eigenvalue that stabilizes at the fold [25]). Thus, the stability of the branches in this simple bifurcation diagram could also be inferred from the (*k*, *d*) diagram (though, in general problems, as discussed in reference [25], different bifurcation diagrams may suggest contradictory stability results). However, the Maxwell construction of equal areas [24] can be applied to the 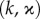 diagram (figure 7*b*) to identify metastable solutions beyond the unstable branch. A more physical take on these stability considerations is the following: under reflection, the (*d*, *k*) diagram maps to the diagram of isotherms of a classical van der Waals gas, for which the middle branch is well known to be unstable [24]. Under this analogy, 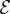 corresponds to the Gibbs free energy of the gas.

This analysis cannot immediately be extended to the more exotic topologies that arise for *λ* close to *λ*_max_ (figure 5). We note however that part of these branches must be unstable, too: as above, a single solution exists for small *d*, and so the corresponding branch must be stable. The first fold must be traversed upwards, and the first branch with 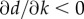 is thus unstable, as above.

An analogous analysis can be carried out for deformations that vary *λ* while keeping *k* fixed: for *k* > k_*_, the (*d*, *λ*) diagram is monotonic, but this ceases to be the case for *k* < k_*_. As above, the stability can be inferred from the (*λ*, *d*) diagram, and the middle branch with 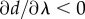 is unstable, too.

The picture that emerges from this discussion is the following: solutions in a region of parameter space underneath the critical point bounded by the spinodal curve are unstable; a band of solutions on either side of this region and below the critical point are metastable (figure 7*c*), both to perturbations varying *k* and to perturbations varying *λ*. Now, as invagination progresses, more cells undergo shape changes [12] (corresponding to *λ* increasing), although the cells are less markedly wedge-shaped at the end of invagination [12], indicating that *k* reaches a maximal value during invagination. The data from thin sections [12] suggest that the bend region does not expand into the anterior hemisphere until later stages of inversion; this corresponds to 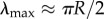 being fixed during invagination. The corresponding paths in parameter space may lead to very different behaviour: if invagination is to be stable, it must move around the critical point; if it passes through the region of in- and metastability underneath, the shell undergoes a subcritical snapping transition between the ‘shallow’ and ‘deep’ invagination states on either side of the metastable region. (This makes this kind of instability different from the classical, supercritical buckling instability of a compressed rod or a ‘popper’ toy [27], and more akin to the snap closure of a Venus flytrap [28].) However, no such snapthrough shows up during invagination in the dynamic data of Höhn *et al.* [19] (the sudden acceleration reported there only occurs after invagination has completed). The present mechanical analysis and these dynamic data thus limit the parameter paths to those that eschew the unstable region underneath the critical point, and reconciles the observed cell shape changes to the stable dynamics: initially, a narrow band of cells undergoes cell shape changes, thereby acquiring a high intrinsic curvature. This region of cells then widens, moving around the critical point, whereupon the preferred curvature relaxes and posterior inversion can complete.

#### 3.2.3. Contraction and criticality

**Figure 8.**
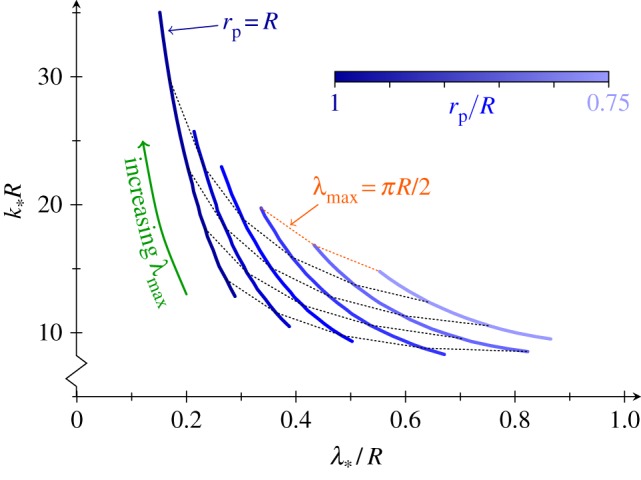
Contraction and the critical point. Trajectories of critical point in parameter space as *λ*_max_ is varied, for different values of *r*_p_. Thin dotted lines are curves of constant *λ*_max_. At constant *λ*_max_, increased contraction leads to decreased k_*_ and increased λ_*_.

For different values of *λ*_max_, the critical point traces out a trajectory in parameter space, characterized by k_*_ and λ_*_ (figure 8). As *λ*_max_ increases, k_*_ increases, whereas λ_*_ decreases. Thus, the closer to the equator, the more difficult invagination is, not only because there is less room to fit the posterior into the anterior, but also because a stable invagination requires narrower, and narrower invaginations of higher and higher intrinsic curvature.

We are left to explore how contraction affects the position of the critical point, and hence the invagination. We introduce a reduced posterior radius *r*_p_ < *R* as in reference [19] (figure 4*a*), and modify the intrinsic curvatures and stretches accordingly (figure 4*b*–*d*). Numerically, we observe that, at constant *λ*_max_, increasing contraction (i.e. reducing *r*_p_) decreases the critical curvature k_*_, and increases λ_*_ (figure 8). Hence, contraction aids invagination not only geometrically, but also mechanically: first, it allows invagination close to the equator (which would otherwise be prevented by different parts of the shell touching), and second, it makes stable invagination easier, by reducing k_*_. Thus, again, contraction appears as a mechanical means to overcome global geometric constraints.

## 4. Conclusion

In this paper, we have explored perhaps the simplest intrinsic deformations of a spherical shell: elastic and geometric effects conspire to constrain deformations resulting from a localized region of intrinsic bending. Contraction, a somewhat more global deformation, alleviates these constraints and thereby facilitates the stable transition from one configuration of the shell to another. This rich mechanical behaviour makes a mathematically interesting problem in its own right, yet this analysis has implications for *Volvox* inversion and wider material design problems.

Experimental studies of *Volvox* inversion [12,19] had revealed the existence of posterior contraction, and indeed, the simple elastic model that underlies this paper can only reproduce *in vivo* shapes once posterior contraction is included [19]. Of course, contraction is an obvious means of creating a disparity in the anterior and posterior radii required ultimately to fit one hemisphere into the other, but the present analysis reveals that, beyond this geometric effect, there is another, more mechanical side to the coin: if contraction is present, lower intrinsic curvatures, i.e. less drastic cell shape changes, are required to stably invert the posterior hemisphere. This ascribes a previously unrecognized additional role to these secondary cell shape changes (i.e. those occurring away from the main bend region): just as the shape of the deformed shell arises from a glocal competition between elastic and geometric effects, a combination of local and more global intrinsic properties allows inversion to proceed stably. Thus, as we have pointed out previously, this mechanical analysis constrains the parameter paths that agree with the dynamical observations of Höhn *et al.* [19] and thereby rationalizes the timecourse of the observed cell shape changes. This lends further support to the inference of Höhn *et al.* [19], that it is a spatio-temporally well-regulated sequence of cell shape changes that drives inversion. Thus, the remarkable process of *Volvox* inversion is mechanically more subtle than it may initially appear to be.

Intrinsic deformations that allow transitions of an elastic object from one configuration to another are of inherent interest in the material design context, and divide into two classes: first, snapping transitions for fast transitions between states, studied in reference [3], and second, stable sequences of intrinsic deformations. The glocal behaviour of the latter is illustrated by the present analysis: in particular, additional transformations such as contraction can increase the number of stable parameter paths between configurations of the elastic object. In this material design context, non-axisymmetric deformations such as polygonal folds or wrinkles [29] could also become important, and may warrant a more detailed analysis.

## Figures and Tables

**Figure 9.**
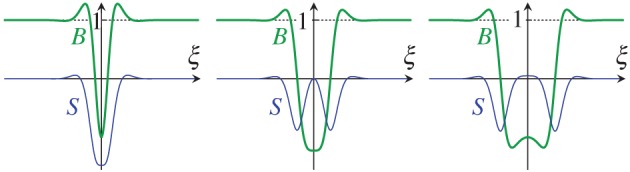
Examples of ‘preferred’ deformation modes, which are solutions of (B 20). Of the modes shown, the middle one has the lowest elastic energy.

**Figure 10.**
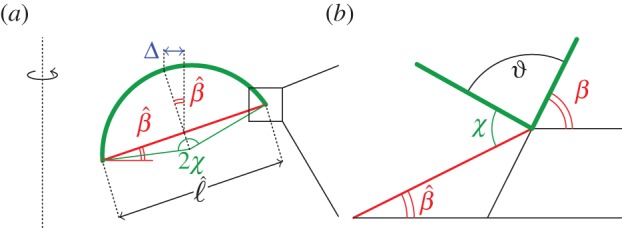
Geometric details in the derivation of the effective energy. (*a*) Hoop strains generated by rotation and inward bending. (*b*) Geometry of formation of second bend region: close-up of the region where the active bend region matches up to the undeformed shell.

## Data accessibility

See www.damtp.cam.ac.uk/user/gold/datarequests.html.

## Appendix A. Governing equations

In this appendix, we sketch the derivation of the Euler–Lagrange equations of the energy functional (2.4), following Knoche & Kierfeld [22]. The variation takes the form

A 1

where we have introduced the stresses and moments

A 2*a*

and

A 2*b*

with  and  (These stresses and moments are expressed here relative to the undeformed configuration.)

The deformed shape of the shell is characterized by the radial and vertical coordinates *r*(*s*) and *z*(*s*), as well as the angle *β*(*s*) that the normal to the deformed shell makes with the vertical direction. These geometric quantities obey the equations [22]

A 3

We note that one of these is redundant. The variations     are purely geometrical, and one shows that [22]

A 4*a*

and

A 4*b*

The variation (A 1) thus becomes

A 5

upon integration by parts, whence

A 6*a*

and

A 6*b*

These equations, together with two of the geometric relations (A 3), describe the shape of the deformed shell. For numerical purposes, it is convenient to remove the singularity at *β* = *π*/2 by introducing the transverse shear tension [20,22],  tan *β*, expressed here relative to the undeformed configuration. Force balance arguments [20,22] show that *Q* obeys

A 7

The solution  is selected by the boundary condition *Q*(0) = 0. At the poles of the shell, the equations have singular terms in them, but these singularities are either removable or the appropriate boundary values are set by symmetry arguments [22]. This allows appropriate boundary conditions and values to be derived.

## Appendix B. Asymptotic details

In this appendix, we discuss the details of the asymptotics summarized in the main body of the paper.

**B.1. Asymptotics of small rotations**

We start by discussing the detailed solution for pure invagination. Upon non-dimensionalizing distances with *R* and stresses with *Eh*, the Euler–Lagrange equations of (2.4), derived in appendix A, can be cast into the form

B 1*a*

and

B 1*b*

with the small parameter

B 2

In these equations, *Σ* is the non-dimensional meridional stress, and *A* = *e_*ϕ*_* is the dimensionless hoop strain. The contribution from the intrinsic curvature is

B 3

The equations are closed by the geometric relation

B 4

Introducing  scaling gives the leading balances   and  in (B 1, B 4). Hence,  and we define an inner coordinate *ξ* via  We also introduce the expansions

B 5*a*

B 5*b*

B 5*c*

This further proves the scaling  that we have assumed in (3.1).

The pure invagination configuration is forced by intrinsic curvature that differs from the curvature of the undeformed sphere in a region of width *λ* about *θ* = *Θ*, where  Writing  we thus have, at leading order,

B 6

where  and where H denotes the Heaviside function. Thus

B 7

where  We note that  provided that  (which we shall assume to be the case); thus, we may set *θ* = *Θ* to the order at which we are working. Expanding (B 1, B 4), we then find

B 8

at lowest order, where dashes now denote differentiation with respect to *ξ*. At next order,

B 9*a*

and

B 9*b*

with  We are left to determine the matching conditions by expanding (3.1) to find

B 10*a*

and

B 10*b*

up to corrections of order  Applying (3.2*b*), at lowest order, we find

B 11

At next order, (3.2*b*) is a system of two linear equations for two integrals, with solution

B 12

In particular, the resulting condition on the leading-order solution has only arisen in the second-order expansion of the matching conditions. Similarly, at order  we find

B 13

The leading-order problem is thus

B 14

with matching conditions (B 11) and the first of (B 12). Symmetry ensures that the first of (B 11) is satisfied. After a considerable amount of algebra, the first of (B 12) reduces to a relation between *K* and *Λ*,

B 15

Symmetry also implies that there is no inward rotation of the midpoint of the invagination at this order. Rather, inward folding is a second-order effect, for which we need to consider the second-order problem,

B 16

with matching conditions (B 13) and the second of (B 12). The rotation of the midpoint of the invagination is thus

B 17

where *B*^(i)^(*Λ*) is determined by the solution of (B 16). The detailed solution reveals that

B 18

The calculation for contraction is similar, but lacks the complication of terms jumping order as above. It is worth noting, however, that the solutions for pure invagination and contraction have the same symmetry at order  and so, in particular, (B 13) is satisfied for both solutions. Hence, the solutions for pure invagination and contraction can indeed be superposed at this order, as claimed in the main text.

**B.2. Asymptotics of large rotations**

In the above analysis, we restricted ourselves to small deviations of the normal angle from the spherical configuration, so that the problem remained analytically tractable. While the leading scaling balances remain the same for large rotations, the resulting nonlinear ‘deep-shell equations’ cannot be rescaled, so that the dependence on *Θ* drops out [21]. Some further insight can, however, be gained in the shallow-shell limit  in terms of the inner coordinate *ξ*, we write

B 19

In the absence of forcing by intrinsic curvatures or stretches, the leading-order balance is

B 20

where dashes denote, as before, differentiation with respect to *ξ*.

This balance also arises in the study of a spherical shell pushed by a plane [21]: at large enough indentations, the shell dimples and the plane remains in contact with it only in a circular transition region joining up the undeformed shell to the isometric dimple. With the matching conditions  as  (B 20) describe the leading-order shape of this transition region [21]. Remarkably, this deformation is independent of the contact force, which only arises at the next order in the expansion [21].

The appropriate boundary conditions for the invagination case are  as  and non-constant solutions of (B 20) can indeed be found numerically (some solutions are shown in figure 9). In these modes, the deformations are, in a sense, large compared with the moments resulting from the intrinsic curvature imposed, making them geometrically ‘preferred’. Their existence lies at the heart of the bifurcation behaviour discussed in the main text.

## Appendix C. Geometric details

In this appendix, we discuss the details of the geometrical approximations that lead to the effective energy discussed in the main body of the paper.

We restrict to the case  in which limit we may approximate  It is most convenient to express the geometric quantities in terms of the angle  intercepted by the deformed bend region (figure 10*a*). By definition,

C 1

and so the leading-order strain balances (3.6), which match the undeformed regions on either side of the active bend region to that region, reduce to

C 2

in this approximation, and the deformed configuration is described in terms of the single parameter *χ* (or, equivalently and implicitly, ).

We now turn to the three physical effects mentioned in the main paper. The curvature in the bend region is  in our approximation, and so the contribution from the preferred curvature of the bend region takes the form

C 3

where the first factor in parentheses corresponds to the area of the undeformed bend region, and where we have discarded numerical prefactors.

The next contribution to the effective energy is somewhat more subtle: as the active region bends inwards, its midpoint moves inwards by a distance Δ (figure 10*a*). Geometry implies that

C 4

The resulting extra hoop strain  constitutes the second contribution to the effective energy

C 5

where, as before, we have neglected numerical prefactors.

The final contribution arises from the second bend region that forms as the active region bends inwards, because the tangents to the rotated active region and the undeformed shell do not match up any longer (figure 10*b*). The difference in tangent angles is

C 6

The size of the resulting bend region is set by the bending lengthscale  of the shell, and is thus, in particular, independent of ℓ. Determining the precise form of the resulting contribution to the energy would require solving the detailed equations describing this bend region. Here, we postulate a simple quadratic form

C 7

where the scale of the prefactor is motivated by the energetics of a Pogorelov dimple [21]. A different power law for the final factor above would not change results qualitatively.
